# Schools of public health as a cornerstone for pandemic preparedness and response: the Africa COVID-19 experience

**DOI:** 10.1186/s12992-024-01087-z

**Published:** 2024-11-21

**Authors:** Rawlance Ndejjo, Honore Kabwebwe Mitonga, Woldekidan Amde, Grace Biyinzika Lubega, Adamson S. Muula, Damen Haile Mariam, Steven N. Kabwama, Sean Mark Patrick, Desderius Haufiku, Maryam Amour, Marc Bosonkie, Trasias Mukama, Segun Bello, Duah Dwomoh, Glory Mbe Egom Nja, Douglas Bulafu, Dabo Galgalo Halake, Gasto Frumence, Mamadou Makhtar Mbacke Leye, Ndasilohenda Katangolo-Nakashwa, Samson Wakuma Abaya, Issakha Diallo, Landry Egbende, Netsanet Worku, Oumar Bassoum, Branly Mbunga, David Musoke, Hussein Mohamed, Ibrahima Seck, Julius Fobil, Suzanne N. Kiwanuka, Olufunmilayo I. Fawole, Mala Ali Mapatano, Tobias Alfven, Lucy Gilson, Jacinta Victoria Syombua Muinde, Harm van Marwijk, Uta Lehmann, Niko Speybroeck, Margaret Kaseje, Rhoda K. Wanyenze

**Affiliations:** 1 Department of Disease Control and Environmental Health, School of Public Health, College of Health Sciences, Makerere University, Kampala, Uganda; 2 School of Nursing and Public Health, University of Namibia, Windhoek, Namibia; 3 School of Public Health, University of the Western Cape, Cape Town, South Africa; 4 Department of Community and Environmental Health, School of Global and Public Health, Kamuzu University of Health Sciences, Blantyre, Malawi; 5 Department of Health Systems Management & Health Policy, School of Public Health, Addis Ababa University, Addis Ababa, Ethiopia; 6 Department of Community Health and Behavioural Sciences, School of Public Health, College of Health Sciences, Makerere University, Kampala, Uganda; 7 School of Health Systems and Public Health, University of Pretoria, Pretoria, South Africa; 8 Department of Community Health, Muhimbili University of Health and Allied Sciences, Dar es Salaam, Tanzania; 9 Department of Nutrition, School of Public Health, University of Kinshasa, Kinshasa, Democratic Republic of Congo; 10 Department of Epidemiology and Medical Statistics, Faculty of Public Health, College of Medicine, University of Ibadan, Ibadan, Nigeria; 11 Department of Biostatistics, School of Public Health, University of Ghana, Accra, Ghana; 12 Department of Public Health, Faculty of Allied Medical Sciences, College of Medical Sciences, University of Calabar, Calabar, Nigeria; 13 School of Nursing and Midwifery, Umma University, Kajiado, Kenya; 14 School of Public Health and Social Sciences, Muhimbili University of Health and Allied Sciences, Dar es Salaam, Tanzania; 15 Department of Preventive Medicine and Public Health, Faculty of Medicine, Pharmacy and Odontology, University Cheikh Anta Diop, Dakar, Senegal; 16 Institute of Public Health, University of Gondar, Gondar, Ethiopia; 17 Department of Environmental and Occupational Health, Muhimbili University of Health and Allied Sciences, Dar es Salaam, Tanzania; 18 Department of Biological, Environmental, and Occupational Health, School of Public Health, University of Ghana, Accra, Ghana; 19 Department of Health Policy Planning and Management, School of Public Health, College of Health Sciences, Makerere University, Kampala, Uganda; 20 Department of Global Public Health, Karolinska Institutet, Stockholm, Sweden; 21 Sachs’ Children and Youth Hospital, Stockholm, Sweden; 22 School of Public Health, University of Cape Town, Cape Town, South Africa; 23 Institute of Health and Society, University of Oslo, Oslo, Norway; 24 Department of Primary Care and Public Health, Brighton and Sussex University Medical School, Sussex, UK; 25 Institute of Health and Society (IRSS), Louvain University (UCLouvain), Brussels, Belgium; 26 Tropical Institute of Community Health and Development, Kisumu, Kenya; 27 Department of Preventive Medicine, School of Public Health, Addis Ababa University, Addis Ababa, Ethiopia; 28 Department of Public Health, School of Allied Health Sciences, Kampala International University, Western Campus, Ishaka, Uganda

**Keywords:** COVID-19, Schools of public health, Africa, Training, Research

## Abstract

**Background:**

The Coronavirus disease (COVID-19) pandemic caused significant morbidity and mortality in Africa, in addition to other socio-economic consequences. Across the continent, Schools of Public Health (SPHs) played several roles in supporting national, regional, and global response to the pandemic. Following a published and grey literature search, this paper reviews and analyses the contribution of SPHs in Africa during the COVID-19 pandemic.

**Contribution of the Schools of Public Health:**

SPH faculty in most countries contributed their expertise through COVID-19 task forces and advisory committees where they guided and supported decision-making. Faculty also supported the identification, review, and synthesis of rapidly evolving global and local evidence, adapting it to the local context to guide policy decisions. Through research, SPHs contributed to a better understanding of the disease epidemiology, response interventions, as well as prevention and control measures. SPHs engaged in training field epidemiologists, frontline health workers, and district response teams. SPH staff, students and field epidemiology trainees also supported field activities including surveillance, contact tracing, as well as managing quarantine facilities and points of entry. SPHs engaged in public education and awareness-raising initiatives to share information and dispel misinformation. In partnership with other stakeholders, SPHs also developed important innovations and technologies.

**Conclusion:**

SPHs are a critical pillar for pandemic prevention, preparedness, and response, that support health systems with important functions. To further enhance their capacity, efforts to improve coordination of SPHs, strengthen collaboration among schools, harmonize training and curricula, and enhance capacity for advanced research are needed. There is also a need to bridge the inequities in capacity and resources that exist among SPHs across regions and countries.

## Introduction

The COVID-19 pandemic caused significant morbidity and mortality in Africa and led to stringent public health and social measures to support the continent’s fragile health systems [1, 2]. Across the continent, the response to COVID-19 resulted in both direct and indirect effects, leading to undesirable health, social, and economic consequences [3, 4]. The pandemic also exposed the vulnerabilities of Africa’s healthcare system and highlighted the urgent need for robust technology and innovations, as well as effective public health systems [5, 6]. The lack of access to personal protective equipment, insufficient training for healthcare workers, and inadequate treatment facilities for infected patients all contributed to undermining the reputation and capabilities of the African countries in responding to the pandemic [7, 8].

As with previous outbreaks, one crucial component of Africa’s COVID-19 pandemic response was the significant role played by academic institutions, notably the SPHs. Within countries, SPHs support training, research, monitoring and evaluation of health systems, and providing services in addition to generating and sharing evidence for decision-making and fostering global health partnerships [9–11]. As centers of excellence in public health, SPHs have historically been a foundation of pandemic and epidemic response, supporting country health systems to deal with Ebola, Marburg, Cholera, Measles, Diphtheria, and Lassa Fever among others [12, 13]. Despite the invaluable importance of SPHs to epidemic preparedness and response, their contribution has often not been comprehensively documented and discussed. Recent literature highlights the role of national public health institutes [14], field epidemiology training programmes [15], actors such as civil society organisations [16] and specific professions. However, the roles and contributions of the SPHs, more so in resource-limited settings, has not received much attention. In this paper, we review and analyse the contribution of SPHs in Africa during the COVID-19 pandemic. By showcasing innovative approaches, research, and capacity-building efforts, we aim to highlight the role of SPHs in preparedness and response to current and future complex public health challenges globally.

## Methods

We searched PubMed and Google Scholar for published articles that discussed contributions to the COVID-19 pandemic response attributed to SPHs. Owing to the limited literature, we developed a reporting template that we shared with members of the SPHs in different countries to provide a summary of their contributions as well as documents and links to country-specific information they were aware of. The template had several sections including the research conducted, response committees engaged in, policy support provided, technology and innovations created, and other roles and contributions made by SPHs in the country. The template was shared widely through the Association of Schools of Public Health network which has a wide membership of faculty across the continent to provide a broader regional picture of the contributions of SPHs in Africa. We extracted relevant information from the published articles, documents and grey literature onto a Microsoft Excel form which we synthesised around relevant themes. The presentation of the themes was supplemented by detailed case studies as shared by the SPHs.

### Contributions of the schools of public health

**Fig. 1:**
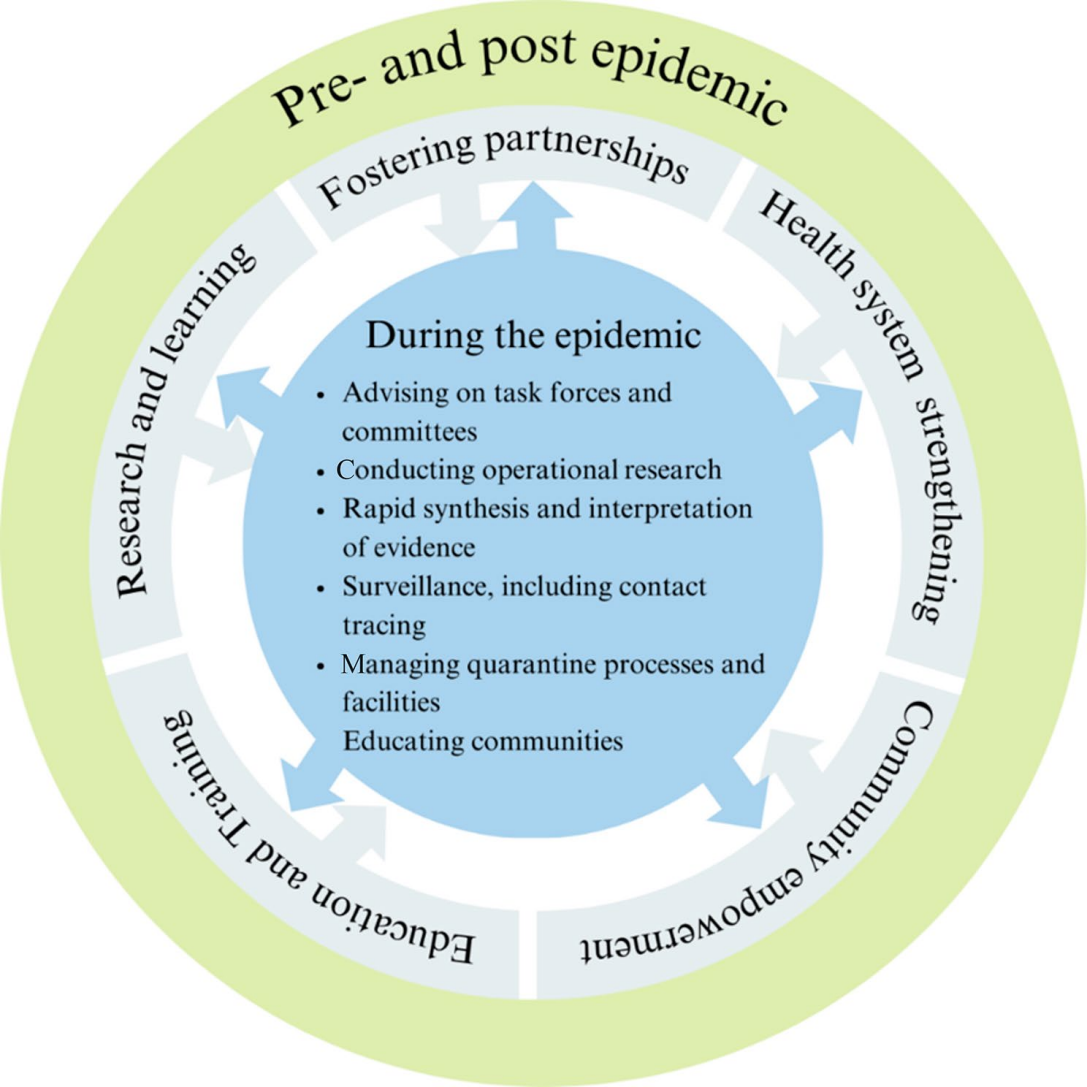
Roles played by SPHs in Africa pre-, during, and post- the COVID-19 pandemic

To support the response to COVID-19, governments worked closely with SPHs especially those within the public universities to support various roles and functions. Most of these SPHs have a clear and close working relationship with the government through their ministries of health where they support public health programming as members of technical working groups, and through research and programme evaluations, evidence synthesis, capacity strengthening, and development of policy guidelines and other documents. On the other hand, the ministries of health contribute to the work of the SPHs through technical expertise, access to resources including data, reports and policies, and support in programme implementation. The SPHs also work closely with local, regional and international organisations through various networks bringing together expertise, mobilising funding, and sharing experiences informing regional policies and practice. Relying on these relationships and amidst the considerable disruptions in routine performance of their functions caused by COVID-19, SPHs continued to perform essential functions to support controlling the outbreak. The roles that SPHs played during the pandemic included: advising task forces and committees, supporting COVID-19 policies at sub-national and national levels, research, training, and supporting field activities such as contact tracing as detailed in the following sections and summarised in Fig. 1.

### COVID-19 task forces and advisory committees

Across the continent, the response to COVID-19 was organized around task forces and committees, most of which were multidisciplinary and multisectoral with varying functions and levels of decision-making. Faculty of SPHs such as in DRC, Nigeria, Ethiopia, South Africa, Senegal, and Uganda were part of Presidential and National task forces overseeing the COVID-19 response in their countries. In some cases, SPH faculty chaired or co-chaired these committees at national, regional, or provincial levels such as in Malawi where a faculty member co-chaired the Presidential Task Force on COVID-19 alongside the Minister of Health. SPHs’ experts were also part of COVID-19 response committees such as the Scientific Advisory Committee, which reviewed the evidence and made policy recommendations to guide the national task forces and the Emergency Operations Centers. Faculty at SPHs also contributed to the core response pillars as they were charged with effective coordination and management of COVID-19 interventions such as Surveillance and Laboratory, Case Management, Infection Prevention and Control (IPC), and Risk Communication. For example, faculty from the Muhimbili University of Health and Allied Sciences SPH in Tanzania were part of the Case Management, IPC & Water Sanitation and Hygiene. In Nigeria, SPH faculty contributed to the IPC, Epidemiology and Surveillance, and Risk Communication Pillars.

### Summarizing evidence and policy support

As one of their key roles, SPH faculty on the COVID-19 response committees identified, reviewed, and synthesised the rapidly evolving global and local evidence and customized it to the local context to inform policy decisions in their respective countries. SPH faculty also supported the review of the performance of various COVID-19 response interventions. These efforts informed key decisions and plans regarding public health and social measures to adopt, their timing and duration, as well as effective treatment regimens. SPHs also worked with ministries of health to translate their data into policies, guidelines, and recommendations. For example, the Universities of Ghana, Makerere, Cape Town, and Nairobi SPHs conducted modelling of the impact of the COVID-19 pandemic and the effect of potential response measures, providing critical information for policymakers to guide decision-making. Researchers at the University of Pretoria significantly contributed to the COVID-19 response by establishing a databank that provided real-time updates and factual information on the pandemic [17]. This initiative aimed to empower decision-makers and the general public with accurate data and statistics through an interactive dashboard, enhancing the understanding of the pandemic’s impact in South Africa. By automating data collection and validation, and offering a user-friendly platform, the group leveraged data science to address social challenges, ensuring resource pooling during crises and promoting transparency to combat misinformation [17]. In Uganda, the national COVID-19 vaccines committee which included three SPH professors was charged with the responsibility of determining which vaccines to procure and expediting their acquisition and distribution across the country [18]. In addition, faculty from the Makerere SPH led the revision of the guidelines for maintenance of essential health services during epidemics in Uganda. The University of Nairobi’s SPH also supported the process of drafting the COVID-19 response bill on behalf of the Kenyan government to protect the rights of citizens during the pandemic.

### Research

SPHs across Africa conducted research to enhance the understanding of the novel SARS-CoV-2 virus and inform policy and interventions within countries and across the continent. For example, SPH researchers in DRC, Ghana, Nigeria, South Africa, and Uganda led and conducted knowledge, attitudes, and practices surveys, rapid assessments, seroprevalence surveys, and studies on the feasibility, safety, and efficacy of potential COVID-19 treatments. The University of Pretoria SPH conducted research on a wide range of themes including genomics, health and socioeconomic consequences of pandemic measures, and vaccine acceptability (Table 1). SPH researchers also conducted operational research to provide real-time evidence to inform the COVID-19 response on the continent across several themes, including the impact of public health and social measures on COVID-19 and non-COVID-19 outcomes, and on health system governance and resilience. Building on their long-standing collaboration, a Makerere University-led partnership of five SPHs in Africa assessed aspects of COVID-19 testing [19], surveillance [20], maintenance of essential health services [21–23], consequences of public health and social measures [24, 25], and uptake of vaccines [26, 27] in the DRC, Ghana, Nigeria, Senegal, and Uganda. Recommendations from this research informed improving coordination of the laboratory network in Nigeria and developing and updating testing and surveillance guidelines for West Africa Health Organization member states. In Uganda, the study findings informed the revision of maintenance of essential health services guidelines [28] and instituting continuity of essential health services as a separate epidemic response pillar. The Addis Ababa SPH in Ethiopia conducted monitoring of hand hygiene, physical distance, and respiratory hygiene in 14 main cities of the country. In Nigeria, the Lagos State University Department of Community Health and Primary Health Care led the COVID-19 daily data update and dissemination in the state for decision-making. Makerere University in Uganda through government funding financed over 110 research and innovation projects, addressing questions critical to the COVID-19 response [29]. SPHs also provided data analysis support to analyse the large volumes of surveillance, contact tracing, laboratory and health facility data collected by the ministries of health. Overall, research across the continent supported improvements in response operations, the deployment of public health and social measures and the rollout of vaccines.

**Table 1:** University of Pretoria’s COVID-19 research contributions

The University of Pretoria School of Health Systems and Public Health (SHSPH) contributed to COVID-19 research by addressing a wide array of challenges presented by the pandemic, evidenced by 53 papers published between 2020–2024 by affiliated staff. The SHSPH explored the complexities of public health genomics in Africa amid COVID-19, offering insights into the adaptation required within healthcare systems to manage pandemic challenges effectively^1^. By investigating the socioeconomic impacts, such as the effects of lockdown measures on residents of South African townships and the socioeconomic inequalities driving child hunger during the pandemic^2^, the SHSPH highlighted the broader economic and health consequences of COVID-19 responses^3^. The SHSPH examination of the pandemic’s impact on mental health services in Sub-Saharan Africa emphasized the critical importance of supporting healthcare workers’ mental health and well-being as an urgent global health priority, showcasing the strain on healthcare professionals during such crises^4^. The research on vaccine acceptability in Africa highlighted the essential factors influencing public trust and participation in vaccination campaigns, which is crucial for the success of such initiatives^5^. Through its comprehensive and multifaceted research efforts, the SHSPH contributed significantly to understanding and addressing the complex challenges of the COVID-19 pandemic, offering insights on pandemic recovery^6^ and suggesting public health strategies and interventions^7^.	

^1^ Moyo E, Moyo P, Mashe T, Dzobo M, Chitungo I, Dzinamarira T. Implementation of Public Health Genomics in Africa: Lessons from the COVID-19 pandemic, challenges, and recommendations. Journal of Medical Virolology. 2023 Jan;95(1):e28295

^2^ Alaba OA, Hongoro C, Thulare A, Lukwa AT. Drivers of socioeconomic inequalities of child hunger during COVID-19 in South Africa: evidence from NIDS-CRAM Waves 1–5. BMC Public Health. 2022 Nov 16;22(1):2092

^3^ Harrigan SP, Tsang VWL, Yassi A, Zungu M, Spiegel JM. Impacts of economic inequality on healthcare worker safety at the onset of the COVID-19 pandemic: cross-sectional analysis of a global survey. BMJ Open. 2022 Oct 5;12(10):e064804

^4^ Dzinamarira T, Iradukunda PG, Saramba E, Gashema P, Moyo E, Mangezi W, Musuka G. COVID-19 and mental health services in Sub-Saharan Africa: A critical literature review. Comprehensive Psychiatry. 2024 Feb 17;131:152465

^5^ Wirsiy FS, Nkfusai CN, Ako-Arrey DE, Dongmo EK, Manjong FT, Cumber SN. Acceptability of COVID-19 Vaccine in Africa. International Journal of Maternal and Child Health and AIDS. 2021;10(1):134–138

^6^ Patrick SM, Cox SN, Guidera KE, Simon MJ, Kruger T, Bornman R. COVID-19 and the malaria elimination agenda in Africa: Re-shifting the focus. Global Public Health. 2022 Dec;17(12):3981–3992

^7^ Chitungo I, Mhango M, Mbunge E, Dzobo M, Dzinamarira T. Digital technologies and COVID-19: reconsidering lockdown exit strategies for Africa. Pan African Medical Journal. 2021 Jun 1;39:93

### Training

SPHs supported the COVID-19 response through training conducted at different levels. In the DRC, Kinshasa SPH trained field epidemiology fellows who were deployed to conduct surveillance and contact tracing. The institution also provided short online courses to frontline health workers to prepare them to receive their first cases of COVID-19 and build their capacity to conduct surveillance. In Nigeria, SPHs alongside field epidemiology graduates and fellows trained health workers on the COVID-19 control pillars (coordination, surveillance, laboratory, and data management) prior to the confirmation of the first case. At the Ibadan SPH, faculty trained rapid response teams at subnational levels about surveillance and IPC. In Uganda, Makerere University initiated a community of practice on IPC for health workers, targeting 2,000 healthcare workers/IPC focal persons from 764 health facilities in 130 districts across the country. The university also trained over 1,000 community health workers (CHWs) to conduct health promotion activities on COVID-19 [30]. Another round of training reached over 900 CHWs and 500 community leaders, with over 19,000 community members vaccinated during the organized COVID-19 vaccination outreaches (Table 2). Makerere SPH together with the College of Veterinary Medicine developed and offered a one-month course on pandemic preparedness following the One Health approach targeting responders, frontline implementers, crisis communicators, social scientists, and agriculturalists [31]. The Tropical Institute of Health’s proactive approach, grounded in community-based data collection and action, extensive training, and strategic collaborations contributed to managing the COVID-19 crisis in Western Kenya. Evidence indicated that the incidence of confirmed COVID-19 infections and deaths was lower in the Institute’s intervention area compared to the control county [32]. The University of Botswana SPH trained cleaning staff at the university on IPC and raised awareness about preventing COVID-19. The other roles SPHs undertook included offering training to media practitioners on COVID-19 interventions and how to communicate decisions to the public. SPHs also offered orientation for district staff on data collection tools and reporting systems.

**Table 2:** Makerere University SPH intervention enhanced uptake of COVID-19 vaccines in Wakiso District, Uganda

Between October 2022 and September 2023, MakSPH implemented a project funded by the Rockefeller Foundation through Amref Health Africa to enhance the capacity of Community Health Workers (CHWs) in Wakiso district to mobilize the community for COVID-19 vaccination and facilitate COVID-19 outreach vaccination campaigns by health facilities. The project also strengthened data management for COVID-19 vaccination. The project trained all CHWs in two Health Sub Districts in the district and supported them to conduct door-to-door health education and community mobilization for COVID-19 vaccination while targeting all households in their communities with unvaccinated members. Local stakeholders including local leaders and Community Based Organisations were also engaged to support community mobilization activities. The project supported health workers to conduct community-based COVID-19 vaccination outreaches and data officers at all public health facilities to ensure that records of all individuals vaccinated were entered into the Ministry of Health COVID-19 vaccination system. The project trained 503 stakeholders (local leaders, religious leaders, and other opinion shapers) who were engaged, and 994 CHWs trained and supported to mobilise community members for COVID-19 vaccination. During the project, a total of 19,384 community members were vaccinated against COVID-19 (8,089 first dose; 3,433 second dose; 7,862 third dose) from 115 vaccination outreaches held (an average of 168 people vaccinated per outreach). Over 2,100 COVID-19 vaccination data entries from earlier campaigns were also made. The project contributed to ensuring a high uptake of COVID-19 vaccination in the most populous district at a time when vaccination rates had been reported to be low.	

### Field support for surveillance and contact tracing

SPHs provided field support to the COVID-19 response. SPH faculty served in the incident command structures where they were deployed to coordinate the regular meetings at national and subnational levels. In Uganda, for example, 26 Master of Public Health students and 25 field epidemiology trainees were part of the COVID-19 workforce conducting surveillance and contact tracing in the community and screening people at points of entry (Table 3). Across countries, staff, students, and field epidemiology fellows also supported rapid response and alert management systems, conducting epidemiological investigations of cases and contacts, managing quarantine sites, and overseeing reporting and line listing. Several faculty of the University of Ibadan SPH including the students of the Field Epidemiology Training Programme supported both state and national emergency operations centres and pillars of COVID-19 response including Epidemiology and Surveillance, Laboratory, and Case Management pillars. Public Health postgraduate students were also deployed to screen international travellers, conduct risk communication with passengers, and monitor persons of interest. In Namibia, field epidemiology and public health (bachelors and masters) students considerably contributed to the establishment of the National Emergency Operations Centre where COVID-19 surveillance activities were performed. The Centre also supported the preparation of integrated support visits to border regions and the strengthening of the coordination and management of response interventions at regional/district levels.

**Table 3:** Makerere University SPH - Master of Public Health alumni and residents supported COVID-19 response in Uganda

Makerere SPH in Uganda deployed 40 front-line epidemiologists from the Master of Public Health (MPH) programme to support the COVID-19 response in Uganda from March 2020. These included 26 MPH alumni volunteers, six current residents, and eight MPH alumni employed by the Uganda Ministry of Health. The Epidemiologists were mostly deployed in Kampala and the surrounding districts where the COVID-19 burden was highest. In April 2020, eight of the MPH alumni volunteers were deployed by the Ministry of Health to support the strengthening of COVID-19 decentralized response. The alumni were deployed in districts with regional referral hospitals to set up rapid response and alert management systems, support epidemiological investigation of cases and contacts, operationalize protocols for sample collection and quarantine site management, and oversee reporting and line lists management. The alumni also supported districts in activating their task force and submitting daily situation reports to the emergency operations centre. Following a rise in the number of cross-border truck drivers that tested positive for COVID-19, another team of 8 MPH alumni and residents were deployed at major Uganda border entry points to set up and activate surveillance team to investigate COVID-19 cases, list their contacts and follow up appropriately.	

### Development of technologies and innovations

SPHs and collaborators contributed to innovations to support the containment of the COVID-19 pandemic. In Uganda, Makerere SPH collaborated with the Ministry of Science, Technology, and Innovation and Kiira Motors Company to develop a low-cost ventilator that can be locally produced [33]. In Nigeria, a collaboration with a non-governmental organization led to the production of hand sanitizers that were donated to the Enugu State Ministry of Health and the University of Nigeria Teaching Hospital [34]. The University of Namibia SPH in collaboration with Namibia Breweries Limited and other stakeholders developed a local hand sanitizer solution initially for university staff and students, but later expanded with private sector interest [35]. The university donated four Philips Respirator Machines to the Ministry of Health and Social Services to support the government’s response to COVID-19. The University of Namibia also introduced a smart phone application for self-reporting of COVID-19 symptoms and another for contact tracing. SPH faculty from the University of Western Cape working with social justice activists and community workers supported the establishment of Community Action Networks which supported and guided communities’ response to the pandemic and associated impacts. Several Community Action Networks were then initiated across Cape Town and were instrumental in dealing with hunger during COVID-19 through initiatives such as community kitchens.

### Public education and awareness

Faculty of SPHs made significant contributions in raising COVID-19 awareness and filling the information void during the response through mass and social media platforms. SPHs such as Ghana SPH developed and disseminated messages to counter misinformation and some provided hotlines for the public to call in. The Tropical Institute of Community Health and Development in Kenya embarked on an extensive community awareness campaign where they trained Community Health Volunteers on preventive measures, equipping them with knowledge and skills to educate the wider community. The Addis Ababa SPH staff educated the public on hand hygiene, physical distancing and respiratory hygiene through a Television program titled “*Tenawo be Bebtewo*” meaning “your health at your house” advocating for obtaining health related information though watching TV. Working with the Tanzania Public Health Association and the World Health Organization, the Muhimbili SPH sensitized 240 older persons on COVID-19 preventive measures in districts in the Kilimanjaro region to enhance their engagement in COVID-19 response. The school also developed information, education, and communication materials for educating the public on COVID-19 prevention and control and held weekly sessions on X (formerly Twitter) spaces. The University of Nairobi SPH through the Elimika Youth Programme provided youth and students with mental health services and information about the pandemic using technology to share facts and reduce misinformation. Across countries including Botswana, DRC, Kenya, Namibia, Nigeria, South Africa, Tanzania, and Uganda, faculty of the SPH frequently conducted newspaper, radio, and television interviews to share information about COVID-19 not only with the general public but with other experts including through opinion pieces and commentaries. These efforts by the SPHs resulted in improved awareness among communities, fostering adherence to preventive measures such as mask-wearing, hand hygiene, and social distancing.

### Other contributions

SPHs also played a critical role in advising and preparing workplaces for reopening during COVID-19 through developing standard operating procedures and training materials, creating a close partnership with the private sector. SPHs convened meetings, conferences, and symposia to share knowledge, expertise and experiences to improve COVID-19 response efforts. The University of Namibia SPH held a global symposia on responses to TB and COVID-19 aiming to improve health and research collaboration. In South Africa, Tanzania, and Uganda, SPHs organized weekly webinars to unite multidisciplinary scientists and facilitate discussions on topical issues about COVID-19. In Malawi, faculty from the Kamuzu University of Health Sciences School of Global and Public Health under the Society of Medical Doctors offered expert opinions in court as amicus curiae (friends of court) when COVID-19 lockdown measures were challenged. Within their universities, faculty of the SPHs provided guidance on standard operating procedures. Several faculty members also engaged with other higher education institutions to develop COVID-19 prevention and control plans. SPH colleagues offered psychosocial support to colleagues and university students.

### Implications

During the COVID-19 pandemic, SPHs in Africa contributed to addressing key gaps in the response and supported local, national, and regional responses. The SPHs undertook critical research to inform policy decisions, supported field activities, trained frontline healthcare professionals on pandemic response, and provided technical assistance to governments and health agencies. Performing these tasks relied on previously formed relationships with the ministries of health, and in undertaking them, they forged new relationships, interactions, and expectations that will influence future programming. On the other hand, working closely with the SPHs lent the government and their ministries of health additional credibility and trust at a time when it was critical, but low, in the public. The collaborative efforts supported buy-in for the response, especially for the implementation of interventions as well as mobilization and lobbying for resources to support the response. Moreover, academic institutions being considered independent, were critical in advocating for evidence-based positions in the policy and decision-making processes. The close working relationship between the government and academia improved transparency and accountability in the response or its perception in most countries. The interaction, however, also came along with potential risks of collective responsibility and accountability with potential repercussions for individual and institutional reputations. In the future, institutions will need to put in place safeguards on how to navigate these relationships to maximise their potential and minimize their negative consequences.

SPHs in Africa bolstered themselves as an important stakeholder in epidemic preparedness and disease response relying on their expertise, legitimacy, and credibility and building on previous efforts and contributions to past outbreaks. This further illustrated the significant role of SPHs in low- and middle-income countries as previously highlighted [10, 36]. SPHs supported national responses to the COVID-19 pandemic by leveraging their expertise as academic institutions. As centres of knowledge generation and evidence synthesis, SPHs guided the policies and interventions that were implemented during the pandemic taking on mostly advisory and strategic roles and in other instances leading important committees. The closer the SPHs were to the decision-making entities, the more prominence and recognition they received amidst more scrutiny and accountability. Both students and faculty contributed directly to the response by training health workers, conducting surveillance and contact tracing as well as engaging in public education. As centres of training, SPHs were a resource for health personnel, directly contributing to the human resource needs during the COVID-19 response. Beyond typical academic roles, SPHs also exerted their expertise to support effective policy and decision-making and strengthen health systems. To achieve these roles, SPHs collaborated with multiple disciplines and stakeholders within and outside of the universities, highlighting the significance, versatility, and interconnectedness of public health with other health and non-health sciences. SPHs also relied on their strong partnerships with the ministries of health, civil society organizations, and other partners, funding from external sources, access to data, abundant skills and expertise, and their pool of trainees to enhance their contribution to the response. The roles played by the SPHs during the pandemic were facilitated by their mandates, functions, and investments. Most of the SPHs also had prior experience supporting previous epidemics and contributing to addressing broader public health challenges in their countries – all of which prepared the SPHs to respond to the COVID-19 pandemic. Amidst the contribution of the SPHs during the COVID-19 pandemic, several challenges existed. The slow and bureaucratic nature of government systems, limited access to funding for activities, the closure of learning institutions, and public misinformation and distrust all impeded the pace of SPHs’ work and minimized their contribution. The fast-changing pace of the pandemic and priorities around it also meant that some SPHs’ work and innovations, even when critical, were not continued and/or sustained.

Although the contributions of SPHs across the continent are presented collectively, countries and regions on the continent experienced the pandemic in different ways which shaped the unique contributions observed and interrogated in the different countries. For example, North and Southern Africa were noted to have had the most devasting COVID-19 impacts [37]. The other factors that impacted the SPHs contributions were related to the level of the country’s development [37, 38], capacity of SPHs including staff expertise and technology access, availability and functionality of national public health institutes [14], existence and strength of field epidemiology programmes [15] and level of collaboration and/or embeddedness with other non-governmental sectors [16]. Moreover, the country’s governance and broader geopolitical context [39] influenced the level and type of engagement with the SPHs. The contribution of SPHs was likely greater in countries that were stable with effective, inclusive, and accountable institutions with a high level of population trust. Whereas COVID-19 negatively impacted globalization [40], it was clear that the collaborative regional networks of SPHs provided avenues for expertise, experience, and resource sharing which impacted country-level response and supplemented government-to-government interaction.

Across SPHs, inequity in capacity and resources exists across regions and countries which impacts their outputs. Indeed, the contribution as presented in this article was not uniform across the continent, countries, and schools. Strong and well-established SPHs situated within public universities tend to be more resourced, networked and more involved in their country’s public health preparedness and response plans. On the other hand, some countries do not have SPHs while others have newly established and under-resourced schools, undermining their contribution to disease prevention and health promotion efforts. It is thus critical to bridge the existing equity gaps and enhance the capacity of SPHs across the continent to conduct training and research, as well as support public health functions. Indeed, with greater capacity and support, the contribution of the SPHs to the COVID-19 response could have been more robust and greater. Strengthened SPHs are crucial to robust and sustainable outbreak preparedness and response. More broadly, well-equipped, and functional SPHs are important to support the continent to deal with the new, emerging, and complex public health challenges that it faces. Indeed, the African continent bears a huge burden of disease influenced by transitions in disease epidemiology, demography, and their dynamic political and socio-economic context [41]. The continent is also prone to epidemics of infectious diseases [42] as well as natural and man-made disasters [43] and bears a large burden of climate change effects [44, 45]. SPHs across the continent thus require dedicated and sustained support from governments, funders, the private sector, non-governmental organizations, and the public to achieve their mandate with sustainability, equity, effectiveness, and efficiency as guiding principles. This is especially critical for the SPHs to play their proactive disease prevention role as opposed to the largely reactionary function whenever extreme events occur. Indeed, whereas a large burden of disease on the continent is preventable, the role of disease prevention and health promotion, paramount for the achievement of universal health coverage and ensuring the health and well-being of the population, is usually given less attention [46, 47]. Other efforts are required to improve the coordination of SPHs, strengthen collaboration among schools, harmonize training and curricula such as for field epidemiologists and public health graduates, and enhance capacity for advanced research. These efforts would position SPHs to undertake their important roles, such as building quality and skilled workforce, and support the health systems on the continent and beyond. The efforts would also contribute to Africa CDC’s new public health order [48] to address structural public health deficiencies and strengthen public health institutions as well as other continental and global agenda.

Whereas this paper has focused mostly on the functions of the SPHs and areas where their contribution was strongest, there were collaborative efforts and contributions from the other health sciences schools and other disciplines across universities. The literature review also focused on sub-Saharan Africa and aspects of response from North Africa may not be represented. The examples highlighted are also not meant to be exhaustive but rather illustrative to provide key insights. We also cannot speak of the effectiveness of the activities implemented which future research should look into.

## Conclusions

Schools of Public Health (SPHs) are a critical pillar for pandemic prevention, preparedness, and response, that support health systems with important functions. During the COVID-19 pandemic, SPHs in Africa leveraged their relationships with the government, local, regional and international collaborations, abundant skills and expertise, and credibility to address key gaps in the response at the local, national, and regional levels. This support was very instrumental, especially in weak health systems of resource-limited settings, positioning them as a cornerstone for pandemic prevention, preparedness, and response and thus should be sustainably supported. However, the contribution of the SPHs was limited within the context of the country’s development, capacity of SPHs including staff expertise and technology access, availability and functionality of national public health institutes, existence and strength of field epidemiology programmes and level of collaboration and/or embeddedness with other non-governmental sectors. To further enhance the contribution of SPHs to pandemic prevention, preparedness, and response efforts are needed to improve the coordination of SPHs, strengthen collaboration among schools, harmonize training approaches and curricula, and enhance capacity for advanced research. There is also a need to bridge the inequities in capacity and resources that exist among SPHs across regions and countries.

## Data Availability

Data is provided within the manuscript.

## Abbreviations

CHWs: Community Health Workers
COVID-19: Coronavirus disease 2019
DRC: Democratic Republic of Congo
IPC: Infection Prevention and Control
MPH: Master of Public Health
SPHs: Schools of Public Health
